# A Theoretical and Practical Treatise on Midwifery

**Published:** 1858-02

**Authors:** 


ART. VII.—*A Theoretical and Practical Treatise on Midwifery: including the Diseases of Pregnancy and Parturition, and the Attention Required by the Child from Birth to the Period of Weaning.* By P. Cazeaux, Member of the Imperial Academy of Medicine, etc., etc. Second American, translated from the Fifth French Edition. By Wm. R. Bullock, M. D. Philadelphia: Lindsay & Blakiston. 1857.

The work which we have before us, has been gradually enlarged, and brought up to the present time in all its particulars, until it now makes a noble volume of one thousand pages, and is certainly the most complete treatise on midwifery and diseases of parturition, in the English language. A translation appeared in 1850, from the second French edition, by Robert P. Thomas, M. D., with which most of our readers are undoubtedly familiar; the present translation is made by Dr. Wm. R. Bullock, from the fifth French edition, the entire work having been carefully revised and a section added on hygiene of children, from the period of birth to weaning; and lastly, "an appreciation of the use of anæsthetics in obstetric practice." The advantages which the French enjoy for the study of this department are very great, and this, combined with the great research and labor which has been brought to bear upon embryology or the function of generation, would lead us to expect in a work intended to embrace all that this work does, a completeness which it is difficult to attain in the other practical departments. For example, in treatises on practical medicine or surgery, it is impossible that the entire ground should be occupied, except in volumes whose ponderous bulk renders them formidable to the brain and purse of the student or practitioner. This object, which it is so desirable to attain in every department, has been most successfully carried out in the present work; and the reader will find, not only a recapitulation of the most rational and best sustained views of those who have attained distinction in this particular field, but he will receive them, when adopted by the author, with the endorsement of practical test which he has been enabled to apply, by his position in lying-in hospitals. By reference to the preface, we see that the author, in acknowledging aid from various sources, is not insensible of the valuable contributions of our own countrymen, Meigs and Dewees.

The length of time during which this work has been before the American profession, and the favorable manner in which it has been received, render a close and analytical review entirely uncalled for; but the great additions which have since been made, and the fact that it has passed into the hands of another translator, render it our duty as well as our pleasure, to present to our readers a slight analysis, and to notice somewhat the important matter which has been added to the first American edition.

The treatise is divided into six parts; beginning in Part first, with the anatomy of the parts concerned in generation and parturition. Of this little need be said. The anatomy of a woman is the same as it was when the first work was written on midwifery, and though of course, it was not as well understood and described as it is at present, yet the parts were there inviting investigation. The facts of anatomy which are disclosed by the scalpel and microscope, are now pretty well established, thanks to the labors of Bichat and his followers; and M. Cazeaux has embodied in his anatomical considerations, all the known facts in relation to this part of the subject. We cannot pass over this section, without referring to the admirable figures with which it is illustrated. The figure representing the uterus and its appendages, is the only one which we have seen, which gives anything like a definite idea of the natural appearances; and this is only one of numerous illustrations to which nearly the same remarks are applicable.

Having concluded the anatomy of the generative organs in Part I, and considered the "modifications undergone by the ovarian vesicles, the concomitant changes of the uterus and the corpus luteum," the author proceeds to a consideration of generation.

Part II then, commences the discussion of generation, beginning with conception, to which some pages are devoted following the changes which take place in the uterus, mammæ, etc., during gestation, embracing the diagnosis of pregnancy, the development of the human ovum, anatomy of the fœtus, and a consideration of the diseases of the pregnant female, uterine displacements, abortion, &c. The space devoted to these subjects is nearly

600 pages. It is not desirable to go fully into an analysis of this section, and we will therefore merely touch upon one or two points where the author seems to advocate some especially useful or original doctrine.

In speaking of the changes which the uterus undergoes during gestation, M. Cazeaux is singularly distinct and conclusive. In the natural state, the neck of the uterus has a spindle-shaped cavity, that is, there is a constriction both at the external and internal mouth, with a dilation in the centre: as pregnancy advances, there occurs ramollissement at the os externum with consequent dilation, so that when the gestation is somewhat advanced, the finger can be admitted, forming instead of a spindle shaped cavity, a cavity like a thimble. The extent to which the finger is able to pass, is a measure of the stage of gestation. During the last few weeks of pregnancy, we have this extending to the os internum, so that now we are able to pass the finger entirely through the neck, and into immediate contact with the membranes. This process of softening and dilation of the os internum goes on, assisted by the feeble contraction of the uterus, until the parturient period; and during this time the neck is never lost or swallowed up in the body of the uterus, as is supposed by some authors, but remains as distinct as ever until the termination of gestation, merely subjected to the changes which M. Cazeaux describes.

The section devoted to the physiology of generation, or the development of the impregnated ovum, is fully up to the times, and may be said to be a complete exposé of all that is known of this important subject. We would recommend this, especially, to the careful perusal of our readers, as we are convinced that they can nowhere find a more complete and satisfactory consideration of the subject of which it treats. We are sorry indeed, that we are not able to follow the author through his admirable descriptions, as this branch of the subject is so interesting to us, that we conceive it could not be uninviting to any one; but we must be content with referring our readers to the work itself, and pass on to Part III.

Part III contains what is justly thought so indispensable to every student and practitioner, who wishes to practice obstetrics intelligently, namely, the mechanism of labor. This subject is here discussed with admirable clearness and simplicity, and commends itself to the careful study of every student of obstetrics. The consideration of the treatment of labor, and the attention to the woman and child necessary immediately after labor, is exceedingly useful, and the latter division especially should be carefully studied by the inexperienced practitioner, as it is on this rock that he often makes shipwreck of his reputation, by allowing the nurse to discover some embarrassment in regard to the disposition of the mother and child immediately after delivery, or a hesitation in making all the necessary prognoses, etc., etc. Though these are really minor points in obstetric practice, yet it is often by attention to these details, that a young doctor begins to establish a reputation as a good practitioner; as the more essential and higher attainments are beyond the comprehension of those who are his judges.

The parts which we have hitherto touched upon have been interesting to us more especially as anatomists and physiologists; but in Parts IV and V we have the consideration of what is most important to us as practitioners of midwifery, namely, the surgery of Dystocia or difficult and unnatural labor.

These treat of every thing which renders labor difficult, and makes it necessary to employ artificial or unusual means in the delivery of the child or the after-birth. They also treat of "the diseases or accidents that may complicate labor, and require the intervention of art:" obstetrical operations, the production of abortion, embriotomy, etc. Of these sections we can only say, that the subjects are handled in the same clear and practical manner, which has characterized those portions of the work, which we have noticed more particularly.

Part VI has been added since the appearance of the first American translation, and treats of the hygiene of children. None of the diseases of children are taken up, but we have about fifty pages devoted exclusively to hygiene, a matter which is so generally neglected, and which is of such great importance. In regard to nursing, M. Cazeaux is decidedly of the opinion that every mother should suckle her own child; and he makes the remark, which strikes us as very just, that there is no necessity of being so strict towards the mother as regards vigor of constitution, quality of milk, and development of breasts, as it is proper to be, in choosing a nurse. "Were we in fact, to regard those women only as capable of nursing, who have the robustness and strength which we require in mercenary nurses, we should be obliged to relinquish the idea of seeing the majority of females in the upper classes suckle their own children. We often find persons of this description who have but little milk, and that of a medium quality, who yet raise very fine children; and what is singular, if these same women should nurse another child, it is found to become emaciated from want of sufficient nourishment." How culpable then, and even criminal, is the unnatural practice of strong and robust women in the upper classes of society allowing a hireling to deprive them of what should be to them a sweet privilege; and how melancholy is it to see the health of a child sacrificed to the selfishness of a mother who is unwilling to forego the pleasure of society, in order to give it its natural nourisnment.

M. Cazeaux is by no means an advocate of the general use of anæsthetic agents in obstetric practice, but he does not go to the extreme lengths in which our countryman, Prof. Meigs, of Philadelphia, indulges. The author presents arguments both for and against their administration, and though he seems to disapprove of them himself, yet the arguments which he adduces, would rather incline to the other side. It is now well established that their administration usually does no harm, and that all we have to fear is the occurrence of an accident, the liabilities to which have been very much magnified. The practitioner should always bear in mind, however, that a great many things are attributed to chloroform or ether, by the friends of a patient, which we know perfectly well are of course in no manner dependent upon it. We have known of cases where death, which did not result until some days after the administration of an anæsthetic in labor, was attributed by the friends to chloroform, and it was said that the doctor killed his patient in this way. A knowledge of these facts should make us cautious, as it is incumbent on us not only to do all the good we can to our patients, but to preserve our own reputation.

In concluding our notice of this work, we do not hesitate to say that it is now the most complete and best treatise on the subject in the English language, and we cordially recommend it as such to our readers- F.

